# Differences in phonetic discrimination stem from differences in psychoacoustic abilities in learning the sounds of a second language: Evidence from ERP research

**DOI:** 10.1371/journal.pone.0187135

**Published:** 2017-11-27

**Authors:** Yi Lin, Ruolin Fan, Lei Mo

**Affiliations:** 1 Center for Studies of Psychological Application & School of Psychology, South China Normal University, Guangzhou, China; 2 Guangdong Key Laboratory of Mental Health and Cognitive Science, South China Normal University, Guangzhou, China; 3 Office of Humanities & Social Sciences, South China Normal University, Guangzhou, China; 4 Institute of Applied Psychology, Guangdong University of Finance, Guangzhou, China; University of Pennsylvania, UNITED STATES

## Abstract

The scientific community has been divided as to the origin of individual differences in perceiving the sounds of a second language (L2). There are two alternative explanations: a general psychoacoustic origin vs. a speech-specific one. A previous study showed that such individual variability is linked to the perceivers’ speech-specific capabilities, rather than the perceivers’ psychoacoustic abilities. However, we assume that the selection of participants and parameters of sound stimuli might not appropriate. Therefore, we adjusted the sound stimuli and recorded event-related potentials (ERPs) from two groups of early, proficient Cantonese (L1)-Mandarin (L2) bilinguals who differed in their mastery of the Mandarin (L2) phonetic contrast /in-ing/, to explore whether the individual differences in perceiving L2 stem from participants’ ability to discriminate various pure tones (frequency, duration and pattern). To precisely measure the participants’ acoustic discrimination, mismatch negativity (MMN) elicited by the oddball paradigm was recorded in the experiment. The results showed that significant differences between good perceivers (GPs) and poor perceivers (PPs) were found in the three general acoustic conditions (frequency, duration and pattern), and the MMN amplitude for GP was significantly larger than for PP. Therefore, our results support a general psychoacoustic origin of individual variability in L2 phonetic mastery.

## Introduction

In the process of learning a new language, we often find that the biggest challenge is the mastery of the foreign accent. Even when we have long contact with a second language (L2) and have been in the L2 environment for a long time, it is hard to distinguish between some non-native language pronunciations. For example, most Japanese native speakers may consider the English words /rock/ and /lock/ to be the same word, because the Japanese speech-sound system does not distinguish between the phonemes /r/ and /l/ [1]. There are two alternative explanations for L2 speech individual differences: a general psychoacoustic (non-speech) origin vs. a speech-specific one.

The general psychoacoustic abilities in early childhood can affect or even predict language acquisition and development [2,3,4,5]. In the acquisition of native language, the ability to process rapid changes of sound is especially vital for early language acquisition [6,7,8,9,10], and the high density analysis of general acoustic features in10-odd milliseconds plays a vital role in analyzing the speech stream and determining the phonetic category. Researchers tend to believe that the general acoustic features in speech must be extracted and analyzed to understand speech information better, such as frequency, duration and presentation order (pattern). And the studies of typical young children [6,11] and children with dyslexia [12,13] have found that the ability to detect, identify and integrate non-speech sounds (included the time characteristics and acoustic spectral characteristics) was very important for processing the general acoustic features in speech and the barriers in the development of language not only derive from the speech-specific system, but also are affected by the general psychoacoustic system. Therefore, the psychoacoustic abilities used to analyze various kinds of acoustic information contained in speech will directly affect the identification of the speech.

What's more, many phonetic training studies [14,15,16] found that faster phonetic learners had stronger psychoacoustic abilities than slower phonetic learners, and they suggested that that individual differences in L2 learning stem from differences in individual general psychoacoustic abilities. Furthermore, that research had explored the neural differences between monolinguals trained to differentiate a difficult Hindi (L2) retroflex phonetic contrast. The studies found that faster phonetic learners appeared to have more white matter in parietal regions, especially in the left hemisphere, and the pattern of results was similar for rapidly changing but not steady-state nonlinguistic stimuli, suggesting that morphological correlates of phonetic learning are related to the ability to process rapid temporal variation [14]. Further studies suggested that left auditory cortex WM anatomy, which likely reflects auditory processing efficiency, partly predicts individual differences in an aspect of language learning that relies on rapid temporal processing [15,16]. The series of studies showed that the acquisition of non-native speech is related to the individual's ability in processing acoustic features; more specifically, the differences in L2 phonetic acquisition stem from the general acoustic processing system.

However, Díaz et al.’s (2008) study [17] showed that the individual variability in L2 phonetic perception is linked to the perceivers’ speech-specific capabilities, rather than the perceivers’ general psychoacoustic abilities. In that study, brain activity of early, proficient Spanish-Catalan bilinguals who differed in their mastery of the Catalan (L2) phonetic contrast /e-ε/ (15 good perceivers vs. 15 poor perceivers) in response to acoustic change detection was recorded in three different conditions involving tones of different length (duration condition), frequency (frequency condition), and presentation order (pattern condition). In addition, neural correlates of speech change detection were also assessed for both native (/o/-/e/) and nonnative (/o/-/o¨ /) phonetic contrasts (speech condition). Participants’ discrimination accuracy, reflected electrically as a mismatch negativity (MMN), was similar between the two groups of participants in the three conditions. Conversely, the MMN was reduced in poor perceivers (PP) when they were presented with speech sounds. Therefore, the results supported a speech-specific origin of individual variability in L2 phonetic mastery. With that evidence, the researchers in that study challenged the theory proposed by the phonetic training studies that differences in phonetic learning stem from differences in psychoacoustic abilities rather than speech-specific capabilities[14,15,16].

Díaz et al.’s (2008) study was very instructive, but close analysis of the research design and the results indicates that the acoustic stimuli materials for pure tone discrimination might not appropriate, which could have led to the null difference between the two groups. First of all, the results of Díaz et al.’s(2008) study showed no difference between groups (GPs and PPs) in the acoustic duration condition, and only the larger deviant stimuli (40ms) elicited a reliable MMN for the GP, whereas the other deviant stimuli (80ms and 120ms) did not elicit reliable MMN. We doubt the results were due to the acoustic stimuli being too difficult for the participants because the participants in Díaz et al.’s (2008) study were Spanish (L1)-Catalan (L2) bilinguals, whose native language is a non-quantity language, which leads to the insensitivity of those bilinguals (both GP and PP) in duration discrimination, and caused the non-significant differences between two groups. Thus, we suggest that if we choose participants whose native language is a quantity language, we may find the differences between GP and PP in duration discrimination.

Secondly, the results in Díaz et al.’s (2008) study showed no difference between groups (GPs and PPs) in the acoustic frequency condition, and only when presented with the larger deviant stimuli (1,090 Hz) a MMN was observed for both groups of participants, whereas the other deviant stimuli (1,030 Hz and 1,060 Hz) did not elicit reliable MMN. We also doubt the results were due to the acoustic stimuli being too difficult for the participants because the duration of the pure tone in the frequency condition was too short to perceive. Thus, we suggest that if we extend the duration of the pure tone in the frequency condition, we may find the differences between GP and PP in frequency discrimination.

Thirdly, the results in Díaz et al.’s (2008) study also showed no difference between groups (GPs and PPs) in the acoustic pattern condition, although, repetition of a tone did elicit a reliable MMN for both groups of participants. We doubt the results were due to the acoustic stimuli being too easy for the participants. Thus, we suggested that if we increase the difficulty of the pattern, we may find the differences between GP and PP in acoustic pattern discrimination.

Accordingly, we chose early, proficient Cantonese (L1)-Mandarin (L2) bilinguals as the participants, because Cantonese (L1) speech includes more acoustic feature information, including tone (齿/ci3/-痴/ ci1/) and duration (夹/gap3/-甲/gaap3/). Compared to the Spanish (L1) participants, the Cantonese (L1) participants may be better in acoustic duration and frequency discrimination. Thus, the Cantonese (L1)-Mandarin (L2) bilinguals in this study were more sensitive to acoustic duration and frequency than Spanish (L1)-Catalan (L2) bilinguals in Díaz et al.’s (2008) study. According to the assumptions of this study, because the acoustic sensitivity of participants was improved in the duration and frequency conditions, the same acoustic frequency and duration stimuli of Díaz et al.’s (2008) study may not cause the same results. If the stimuli were still too difficult, we could change the parameters to decrease the difficulty, in order to explore the differences between GP and PP. Conversely, in the pattern condition we would increase the difficulty of stimuli appropriately, in order to explore the differences between GP and PP.

This study adopted the same experimental procedures used in Díaz et al.’s (2008) study: Firstly, we explored participants’ capacity to perceive a difficult L2 contrast. Then, we selected and divided them into two groups (good perceivers and poor perceivers) according to their performance on a phonetic discrimination task. After the behavioral task, participants’ MMN was measured to evaluate the neural correlates of acoustic stimuli discrimination.

MMN can reflect the individual ability to discriminate differences between acoustic stimuli as they occur after an infrequent change in a repetitive sequence of sounds (sometimes the entire sequence is called an oddball sequence). In the oddball paradigm, the stimuli is divided into two categories, standard and deviant stimuli, and the MMN is elicited when the auditory perceptual system detects a mismatch between a neural representation of a frequently repeated stimulus (the standard) and a stimulus deviating in at least one parameter (the deviant). This ERP component peaks between 100–250ms. It should be noted that the amplitude of the MMN is directly related to the magnitude of the perceived change and, hence, it is considered a measure of individual auditory discrimination accuracy. The results of many previous studies showed that MMN is sensitive to changes both in tones [18,19] and speech sounds [20,21,22,23]. Moreover, MMN often appears in the fronto-central area, and the amplitude in the prefrontal area is negative and the largest. Meanwhile, there will be a positive reversed MMN appearing in the temporal area.

Therefore, it is supposed that the neural activity underlying the MMN is attributable to two sets of neural generators: a superior temporal and a fronto-central generator. The former is associated with processing the auditory sensory input against a formed memory trace, whereas the latter is related to an involuntary attention switch toward a detected change in the auditory input [24,25]. Thus, if the GP and PP do show differences in processing pure tone, we would like to identify which processing region the differences come from.

In a word, in this study we attempt to explore the relationship between the general psychoacoustic abilities and the phonetic discrimination in the second language, by choosing the proper participants and properly adjusting the parameters of the previous research[1].

## Experiment

In order to identify the neural correlates of general psychoacoustic abilities and phonetic discrimination in perceiving L2 phonetic contrasts, there are three experiments were designed to assess general acoustic discrimination accuracy in three conditions in which pure tones were presented: duration, frequency, and pattern conditions.

### Purpose

The purpose of experiment 1 was to examine whether the good perceivers group and poor perceivers group were different in perceiving the duration of pure tone. Díaz et al.’s (2008) study showed that in the duration condition, only the larger deviant stimuli (40ms) elicited a reliable MMN for the GP group and a marginal MMN for the PP group, but there was no difference between groups, and the interaction was not significant. This may be because the duration of materials was too difficult to distinguish for Spanish speakers. So in our study, we recruited Cantonese speakers who have stronger ability to distinguish the duration of tone as participants in order to reveal the differences between the GP and PP groups in perceiving the duration of pure tone.

The purpose of experiment 2 was to examine whether the GP and the PP groups were different in perceiving the frequency of pure tone. Díaz et al.’s (2008) study showed that in the frequency condition, a reliable MMN for both groups of participants was evident only when presented with the larger deviant stimuli (1,090 Hz), with no significant difference between groups or any group factor interactions, which may also be due to the stimuli were too difficult to distinguish. In this study, we adjusted the difficulty of the materials for identifying the frequency of pure tone to reveal the differences between good perceivers and poor perceivers in perceiving the frequency of pure tone.

The purpose of experiment 3 was to examine whether the GP group and the PP group were different in perceiving the pattern of pure tone. Díaz et al.’s (2008) study showed that in the pattern condition, repetition of a tone elicited a reliable MMN for both groups of participants. However, there were no significant differences between groups, which may have been due to the stimuli were too easy to distinguish. In this study, we increased the difficulty of the materials for identifying the pattern of pure tone to reveal the differences between the GP group and the PP group in perceiving the pattern of pure tone.

### Method

The study protocol was approved by the South China Normal University Research Ethics Committee, and the research was conducted in accordance with the ethical standards specified in the 1964 Declaration of Helsinki and its later amendments. Written informed consent was obtained from the participants, and they could freely withdraw from the study at any time.

#### Participants

The selection of participants was the key to this study. All participants were undergraduate students from the South China Normal University. The process was divided into three steps. In the first step, we enrolled 130 early, proficient Cantonese (L1)-Mandarin (L2) bilinguals (mean age 24.4±1.8) in the university from non-language majors. As requested, all of the bilinguals were born in the Cantonese environment, since the age of 3 or 4 in the kindergarten, they had begun to learn Mandarin, before that time they could only communicate in Cantonese, and from that time on, they had been living in the Cantonese-Mandarin bilingual environment. In the meantime, 28 healthy native Mandarin (L1) speakers (mean age 25.2±1.6) who were born and grew in the pure Mandarin environment were selected as the standard Mandarin group, and their performance in the Mandarin phonetic discrimination test was used as a standard to filter the GP and PP groups of participants. The 28 native Mandarin (L1) speakers were from Hebei, Jilin, Heilongjiang, Beijing and Liaoning, which are pure Mandarin language regions.

In the second step of participant selection, the GP and PP groups of participants were chosen from the 130 Cantonese (L1)-Mandarin (L2) bilinguals by a phonetic discrimination test. Mandarin syllables consist minimally of an initial, a final, and tone. According to the phonological features of Mandarin and the interview with several senior Chinese teachers, in the pronunciation, nasal finals /in / and / ing/ are the most difficult to distinguish. In another word, the phonetic contrast / in/—/ ing / can identify whether a person is good Mandarin speaker. And all the participants agreed that /in/-/ing/ contrast is the most difficult phonetic contrast to discriminate both in listening and speaking, especially for the Cantonese native speakers. Therefore, this study adopted a behavioral task (lexical decision task) [26] to assess the bilingual participants’ ability to identify /in / and / ing/. Vocabulary words for testing were 144 Mandarin two-character words. Half of the words contained the final sound /in/, whereas the other half contained the final sound /ing/. Half were real words, and half were pseudo words. The pseudo words were created by altering the true words. That is, the pseudo words were created by changing the true pronunciation of /in/ into the /ing/ pronunciation or vice versa. For example, the pseudo word [ying1gao1] was changed from the true word "音高" [yin1gao1], or conversely, the pseudo word [huo3xin1] was changed from the true word "火星" [huo3xing1]. Participants were asked to judge whether the words were true words or pseudo words by listening to them. All of the 130 Cantonese (L1)-Mandarin (L2) bilinguals and the 28 native Mandarin (L1) speakers completed this test.

The accuracy rates of all of the participants were changed into $\bar{A}$ scores [27], which reflected the subjects’ ability to discriminate words, that is the ability of correct lexical representation according to Mandarin phonetic contrast (/ in/—/ ing /). The transformation of $\bar{A}$ scores is a statistical method based on signal detection theory. $\bar{A}$ scores are calculated according to the participants’ hit rate P(S | s) and false alarm rate P(S | n) in the task. The computation formula is as follows, where x = P(S \ s) and y = P(S \ n):

$$P\left(\bar{A}\right)=(\frac{1}{2}(\frac{x^{3}+x^{2}(y-2)-x(y+3)(y-1)-y{\left(y-1\right)}^{2}}{2x(1-y)})$$

Results showed that the accuracy of Cantonese (L1)-Mandarin (L2) participants identifying /in/-/ing/ was very low, with an average $\bar{A}$ score of 0.67 (SD = 0.11). The native Mandarin (L1) speakers had higher accuracy, with an average $\bar{A}$ score of 0.89 (SD = 0.062). There were 16 bilinguals (12.3%) assigned to the GP group because their $\bar{A}$ scores were in plus or minus three standard deviations of the Mandarin (L1) speakers’ average $\bar{A}$ score. The GP group’s average $\bar{A}$ score was 0.87 (SD = 0.04). Correspondingly, there were 16 bilinguals (12.3%) whose scores were at the bottom assigned (much lower than minus three standard deviations of the Mandarin (L1) speakers’ average $\bar{A}$ score) to the PP group. The PP group’s average $\bar{A}$ score was 0.61 (SD = 0.05). The difference between the two groups was significant (p<0.001).

In the third step of participant selection, the final participants were filtrated according to the requirements of the ERP experiment. Through the above test, 16 GP participants and 16 PP participants were selected. However, there were two left-handed participants and two EEG-abnormal participants who had to be ruled out. Thus, each of the two groups was left with 14 participants. The GP group consisted of 11 females and 3 males. The PP group consisted of 13 females and 1 male. The difference of the $\bar{A}$ score between the two groups was significant (p<0.001).

Participants received a small payment for their participation. They all signed the corresponding written informed consent form. All participants were right-handed, and none of them reported having any hearing or language difficulties or receiving specific musical training.

#### Procedure and materials

In experiment 1, we used the same sound stimuli as in Díaz et al.’s(2008) study. In the duration condition, the stimuli were pure tones of 1,000 Hz. The duration of the standard tone was 200ms (including 10ms of rise/fall times) and the durations of the three deviant tones were 120, 80, and 40ms.

The probability of the standard tone was always 0.8 (600 standard tones per block), and for each deviant tone the probability was 0.066 (50 presentations of each deviant tone per block). Tones were presented in random order with the restriction that the first five tones of the blocks were always a standard and that at least one standard tone was presented between two deviants. The stimuli onset asynchrony (SOA) was 314ms.

In experiment 2, we also used the same sound stimuli materials as in Díaz et al.’s (2008) study. In the frequency condition of Díaz et al.’s (2008) study, the auditory stimuli were pure tones of 50ms (including 10ms of rise/fall times). The frequency of the standard tone was 1,000 Hz, whereas the frequencies of the deviant tones were 1,030, 1,060, and 1,090 Hz. The probability of the standard tone was always 0.8 (600 standard tones per block), and for each deviant tone the probability was 0.066 (50 presentations of each deviant tone per block). Tones were presented in random order with the restriction that the first five tones of the blocks were always a standard and that at least one standard tone was presented between two deviants. The SOA was 314ms. In this study, the auditory stimuli duration was increased from 50ms to 100ms (including 10ms of rise/fall times) to properly reduce the difficulty of discrimination.

In experiment 3, we partly used the same sound stimuli materials as Díaz et al.’s (2008) study. In the pattern condition of Díaz et al.’s (2008) study, 400 trains of tones were presented. Each train consisted of six alternating pure tones of either 500 or 1,000 Hz (2,400 tones altogether). Tones lasted 50ms, including 10ms rise/fall times. Tones within and between the trains were presented at a constant SOA of 128ms. Stimuli trains were presented in a predictable way (ABABAB-BABABA-BABABA-ABABAB…), in which A represents the 500 Hz tone and B the 1,000 Hz tone, the hyphen indicates the beginning of the trains, and the bolded A and B denote the deviant event. In other words, in some cases, the presentation pattern was violated by repeating one of the two tones. In that case, repetition of a tone was the deviant stimuli. We adopted the same pattern as Pattern 1 in this study.

Furthermore, we developed a Pattern 2 to increase the difficulty of the materials, based on Pattern 1. Pattern 2 also consisted of 400 trains of tones, and shared the same duration, frequency, and constitution with Pattern 1. Each train in Pattern 2, however, consisted of four alternating pure tones, and the stimuli trains were presented in a predictable way (ABAB-BABA-BABA-ABAB…). The repetition of a tone was the deviant stimuli here as well.

During the EEG recording, participants were in an electrically shielded soundproof room while watching a silent movie. All of the stimuli were delivered binaurally through headphones, but participants were asked to concentrate their attention on the movie and to ignore auditory stimulation. There were five films for participants to choose from.

#### Electrophysiological recording and ERP data analysis

The experimental apparatus used to continuously acquire EEG recordings was the Neuroscan 32-channel SynAmps2 amplifier. The ERPs were recorded from the scalp by using tin electrodes mounted in an electrocap (Electro-Cap International) and located at six standard positions (F3, Fz, F4, C3, Cz, and C4) and the two mastoids (LM and RM). The eight electrode points covered two placing signage (fronto-central and supratemporal lobe) of MMN. Eye movements were measured with electrodes attached to the infraorbital ridge and on the outer canthus of the right eye. The common EEG/electrooculogram (EOG) reference was attached to the tip of the nose. Electrode impedances were kept <5kΩ. The electrophysiological signals were filtered on-line with a bandpass of 0.1–100 Hz and digitized at a rate of 1000 Hz. We used Neuroscan as the EEG data processing software to process off-line data. First, the influence of the EOG was removed. Then, epochs with EEG exceeding either ±70μV at any channel were automatically rejected off-line. Epochs included in all cases a prestimuli baseline of 100ms and were 600ms long. Baseline was corrected, and the linear DC detrend procedure was performed on the individual segments. Individual ERPs were digitally band-pass filtered between 0.8–20 Hz. Lastly, brain electrical signals induced under various experimental conditions were superposed and averaged respectively.

The MMN was identified using the difference between waveforms obtained by subtracting the standard ERPs from those elicited by deviant stimuli. The MMN was measured for each participant group and condition as the mean amplitude in a 20ms latency window centered at its maximum peak. To test whether a significant MMN was elicited by deviant stimuli, one sample t-tests were carried out (separately for each group of participants) to compare the amplitudes of the MMN component at Fz against the zero level [1]. A repeated-measures ANOVA was performed for each condition separately. Only deviants eliciting a reliable MMN at least for one group were included in the analysis. In this step, the positive MMN for the supratemporal lobe had to be reversed into negative MMN with the same absolute value. The factor’s laterality (left hemisphere: F3, C3, LM; right hemisphere: F4, C4, RM), frontality (frontal location: F3, F4; central location: C3, C4; supratemporal location: LM, RM), and deviant type (when necessary) were included in the ANOVA as within subjects factors, whereas participant group (GPs and PPs) was the between subjects factor. Significance levels of the F ratios were adjusted with the Greenhouse–Geisser correction, and the corrected p-values are reported.

### Results and analysis

#### Experiment 1

**Fig 1:**
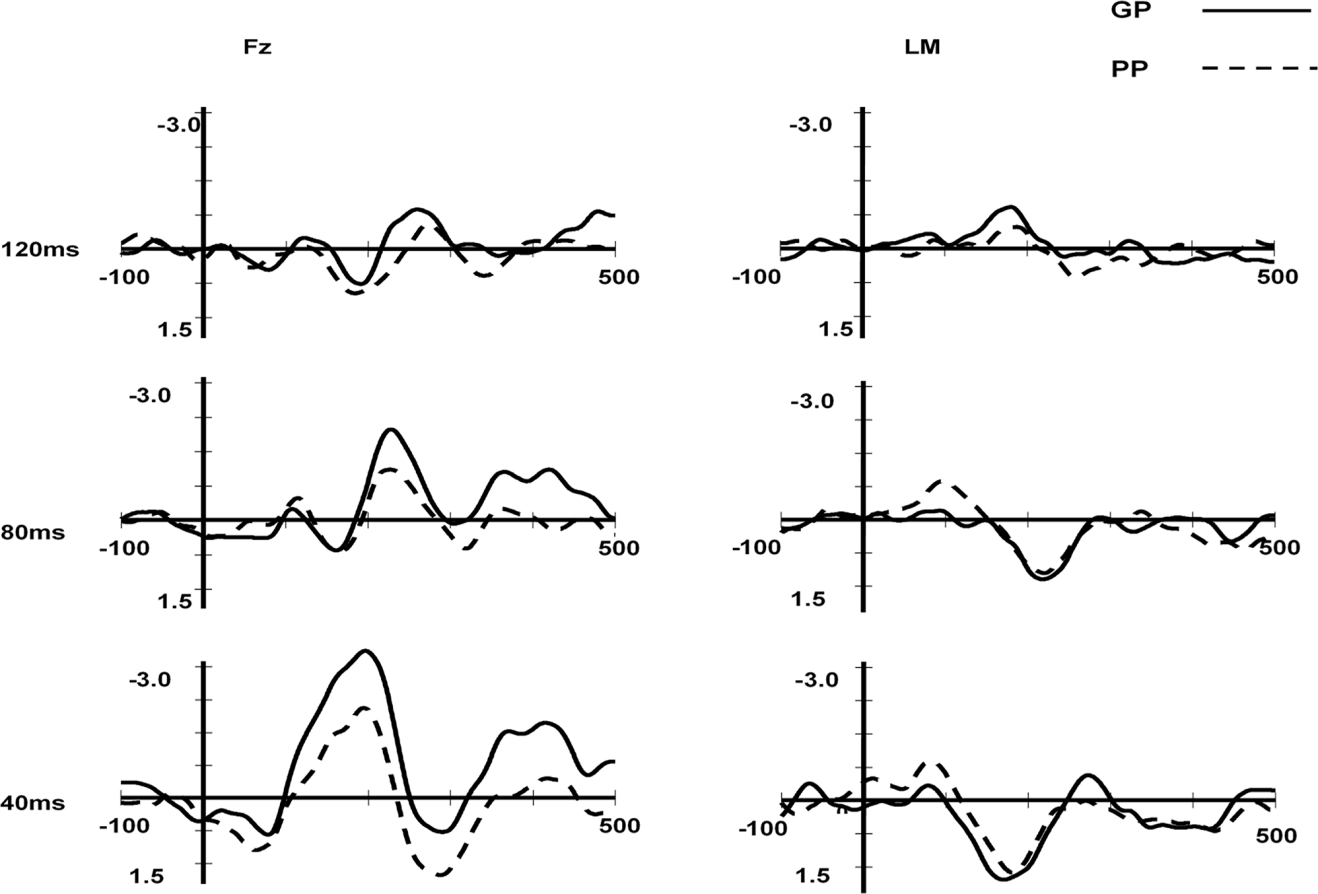
MMN obtained for the GP and PP groups in the duration condition at the Fz and LM electrode.

To test whether the deviant stimuli induced significant MMN across the three different pure tone durations (120, 80, and 40ms), the amplitudes of the MMN component at the frontal Fz electrode were compared with the zero level separately for each group of participants (GPs and PPs; see Table 1 and Fig 1).

**Table 1:** The t test of the MMN mean amplitude for the duration conditions at Fz.

		Good perceivers			Poor perceivers		
Duration	Latency window, ms	Mean amplitude,μV	t	df	Mean amplitude,μV	t	df
120ms	246–286	-0.79	3.80**	13	-0.37	1.29	13
80 ms	207–247	-1.79	3.76**	13	-1.18	5.19**	13
40 ms	175–215	-3.23	9.68**	13	-1.87	5.95**	13

Significant differences

**, P<0.01. df, degrees of freedom.

Table 1 and Fig 1 show only that the PP group did not obtain statistically reliable MMN in the duration condition of 120ms deviant stimuli at the Fz electrode. Thus, a 2 (phonetic discrimination: GP, PP) × 2 (laterality: left hemisphere, right hemisphere) × 3 (frontality: frontal, central, supratemporal) ANOVA was performed for the three deviant stimuli conditions (120ms, 80ms, 40ms) separately. The variance analysis results showed three things. First, none of the main effects or interactions were significant when the deviation stimulus was 120ms. Second, only the main effect of frontality was significant (F(2,26) = 5.25, p = 0.022<0.05, frontal = -1.32μV, central = -0.61μV, supratemporal = 1.17μV) when the deviation stimulus was 80ms, whereas the other main effects and interactions were not significant. Third, the three main effects were significant when the deviant stimulus was 40ms, but the interactions were not. The main effect of discrimination ability (F(1,26) = 8.75, p<0.01, GP = -2.30μV, PP = -1.47μV) showed that the MMN amplitude for the GP group was larger than for the PP group. The main effect of frontality (F(2,26) = 4.53, p = 0.033<0.05, frontal = -2.43μV, central = -1.73μV, supratemporal = 1.51μV) showed that the MMN amplitude for the supratemporal lobe was larger than for the frontal and central lobes. The main effect of laterality (F(2,26) = 13.93, p<0.01, left = -1.74μV, right = -2.03μV) showed that the right hemisphere had an advantage over the left.

It is notable that the results of Díaz et al.’s(2008) study showed no difference between groups (GPs and PPs) in the duration condition, and only the larger deviant stimulus (40ms) elicited a reliable MMN for the GP group, whereas the other deviant stimuli (80ms and 120ms) did not elicit a reliable MMN. However, in this study, the deviant stimuli 40ms and 80ms both elicited a reliable MMN for the GP and PP groups, and the MMN amplitude for GP was larger than for PP when the deviant stimulus was 40ms. The difference between the two studies suggests that the tonal duration discrimination abilities of Cantonese (L1)-Mandarin (L2) bilinguals are better than Spanish (L1)-Catalan (L2) bilinguals. The results of this study support that participants who differed in their L2 phonetic discrimination had different general psychoacoustic abilities in duration discrimination. Díaz et al.’s (2008) study showed no difference between the two groups (GPs and PPs) in the duration condition because the experimental materials for Spanish speakers were not appropriate. The current study indicated that the GP group was better than the PP group in perceiving general acoustic duration when the deviant stimulus was 40ms, which complied with the assumptions of this research.

#### Experiment 2

**Fig 2:**
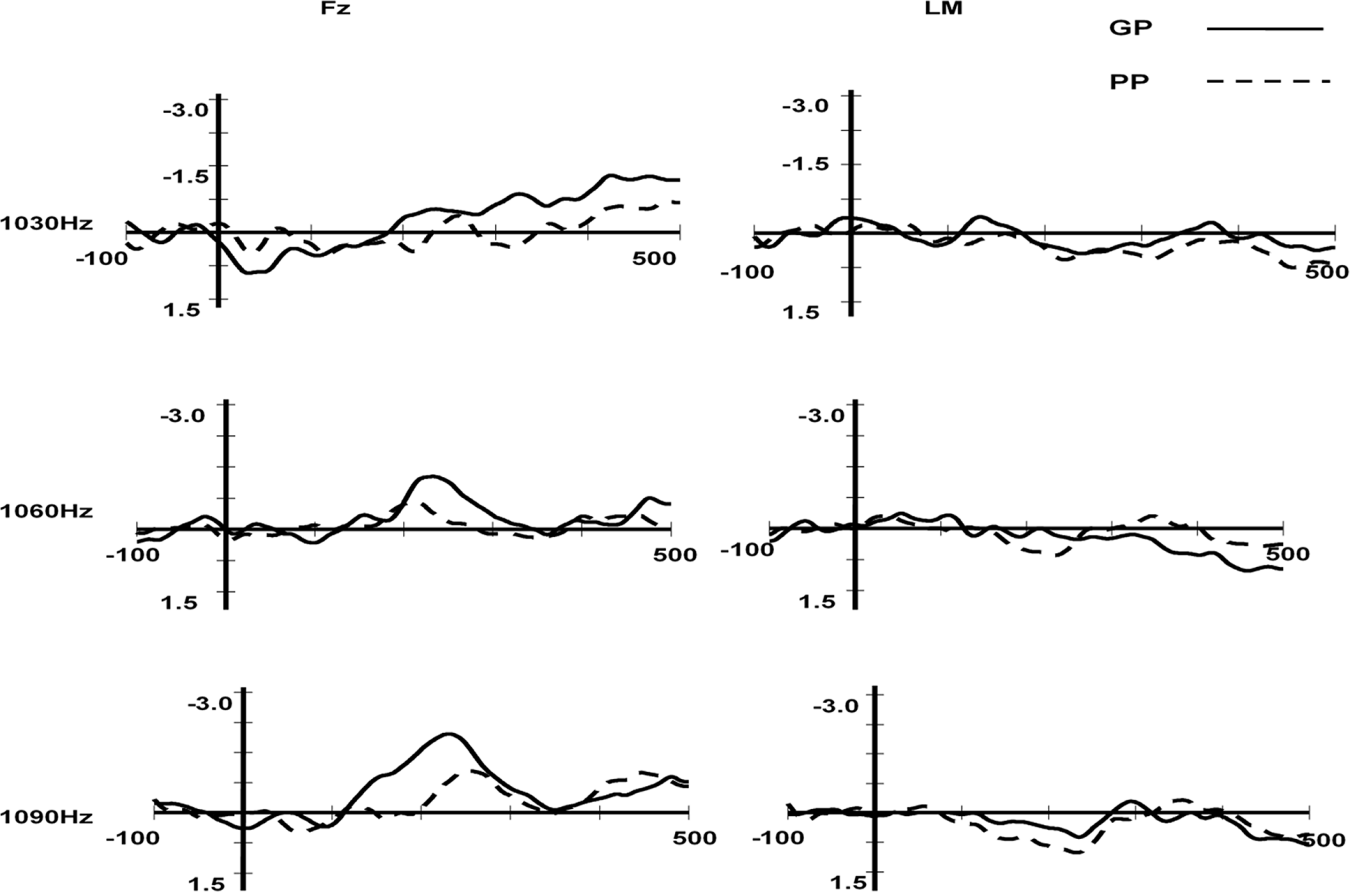
MMN obtained for the GP and PP groups in the frequency condition at the Fz and LM electrode.

To test whether the deviant stimuli induced significant MMN in the conditions of three different pure tone frequencies (1,030, 1,060, and 1,090 Hz), the amplitudes of the MMN component at the frontal Fz electrode were compared with the zero level separately for each group of participants (GPs and PPs; see Table 2 and Fig 2).

**Table 2:** The t test of the MMN mean amplitude for the frequency conditions at Fz.

		Good perceivers			Poor perceivers		
Frequency	Latency window, ms	Mean amplitude,μV	t	df	Mean amplitude,μV	t	df
1,030 Hz	227–267	-0.50	1.65	13	-0.22	0.57	13
1,060 Hz	213–253	-1.22	4.03**	13	-0.42	1.20*	13
1,090 Hz	221–261	-1.81	7.68**	13	-0.91	4.84**	13

Significant differences

*, P<0.05

**, P<0.01. df, degrees of freedom.

Table 2 and Fig 2 show that the GP and PP groups did obtain statistically reliable MMN in the frequency conditions of 1,060 and 1,090 Hz deviant stimuli at the Fz electrode, but they did not obtain statistically reliable MMN in the frequency condition of 1,030 Hz deviant stimuli. Thus, a 2 (phonetic discrimination: GP, PP) × 2 (laterality: left hemisphere, right hemisphere) × 3 (frontality: frontal, central, supratemporal) ANOVA was performed for the two deviant stimuli conditions (1,060 and 1,090 Hz) separately. The variance analysis results showed two things. First, when the deviation stimulus was 1,060 Hz, the interaction of frontality and phonetic discrimination was significant (F(1,26) = 4.82, p = 0.029<0.05). After simple effect inspection, we found that the MMN amplitude for the GP group was larger than for the PP group at the frontal location (t(13) = 2.20, p = 0.037<0.05, GP = -1.07μV, PP = -0.44μV). The difference between the MMN for the GP and PP groups was marginally significant at the central location (t(13) = 1.87, p = 0.07, GP = -0.82μV, PP = -0.13μV), and not significant at the supratemporal location. The other main effects and interactions were not significant. Second, when the deviant stimulus was 1,090 Hz, the main effect of phonetic discrimination was significant (F(2,26) = 6.27, p = 0.019<0.05, GP = -1.19μV, PP = -0.75μV). The main effect of frontality was also significant (F(2,26) = 4.02, p = 0.05), with the MMN amplitude for the frontal lobe being larger than the supratemporal and central lobes (frontal = -1.35μV, central = -0.86μV, supratemporal = 0.71μV). The main effect of laterality was also significant (F(2,26) = 7.66, p = 0.01), with the right hemisphere having an advantage over the left (left = -0.88μV, right = -1.06μV). The interaction of frontality and phonetic discrimination was significant (F(2,26) = 5.83, p = 0.02<0.05). After simple effect inspection, we found that the MMN amplitude for the GP group was larger than for the PP group at the frontal location (t(13) = 3.13, p<0.01, GP = -1.78μV, PP = -0.91μV). Also, the MMN amplitude for the GP group was larger than for the PP group at the central location (t(13) = 2.66, p = 0.013<0.05, GP = -1.32μV, PP = -0.39μV). The difference in the MMN between the GP and PP groups was not significant at the supratemporal location.

The results showed that the GP group was significantly better than the PP group in perceiving pure tone frequency after extending the duration of the pure tone, and the advantage of the GP group was more evident at the frontal and central lobes. Thus, the results of this study support that participants who differed in their L2 phonetic discrimination had different general psychoacoustic abilities in frequency discrimination. Díaz et al.’s (2008) study showed no difference between the two groups (GPs and PPs) in the frequency condition, and only when presented with the larger deviant stimuli (1,090 Hz) a MMN was observed for both groups of participants, whereas the other deviant stimuli (1,030 Hz and 1,060 Hz) did not elicit reliable MMN. The results might because the experimental materials for Spanish speakers were too difficult (the duration of the pure tone in the frequency condition was too short to perceive). This study indicated that the GP group was better than the PP group at perceiving general acoustic frequency when the deviant stimuli were 1,060 and 1,090 Hz, after we lowered the difficulty of the materials, which complied with the idea of this research.

#### Experiment 3

**Fig 3:**
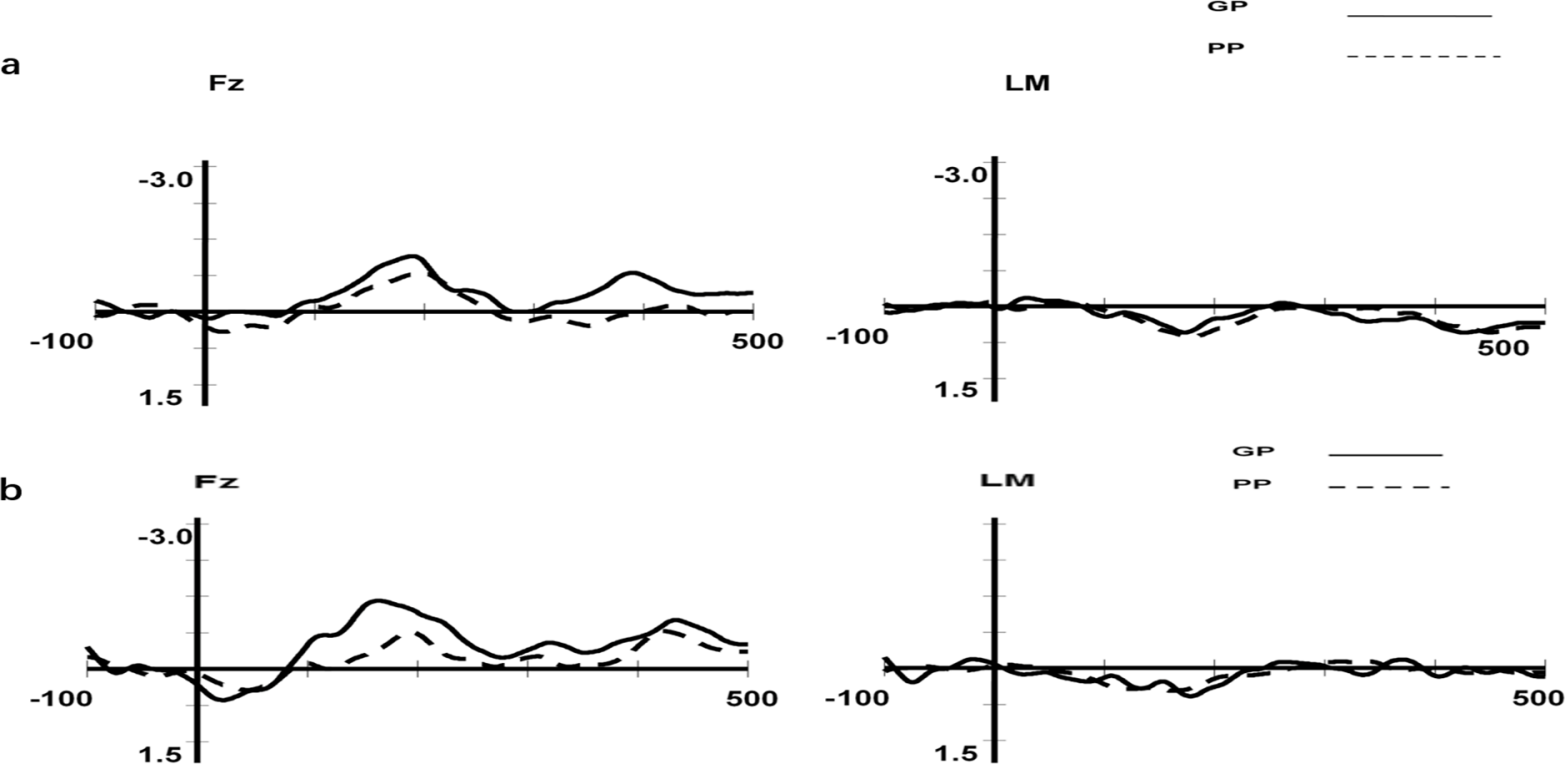
MMN obtained for the GP and PP groups in the pattern condition at the Fz and LM electrode. Fig 3(a) shows the MMN obtained for the GP and PP groups in the pattern1 condition at the Fz and LM electrode. Fig 3(b) shows the MMN obtained for the GP and PP groups in the pattern2 condition at the Fz and LM electrode.

To test whether the deviant stimuli induced significant MMN in the condition of two different pure tone patterns, the amplitudes of the MMN component at the frontal Fz electrode were compared with the zero level separately for each group of participants (GPs and PPs; see Table 3 and Fig 3).

**Table 3:** The t test of the MMN mean amplitude for the pattern conditions at Fz.

		Good perceivers			Poor perceivers		
Condition	Latency window, ms	Mean amplitude,μV	t	df	Mean amplitude,μV	t	df
Pattern 1	173–213	-0.97	5.44**	13	-0.73	3.72**	13
Pattern 2	159–199	-1.27	6.14**	13	-0.66	3.00**	13

Significant differences

**, P<0.01. df, degrees of freedom.

Table 3 and Fig 3 show that the GP and PP groups did obtain statistically reliable MMN in the condition of Pattern 1 and Pattern 2 at the Fz electrode. Thus, a 2 (phonetic discrimination: GP, PP) × 2 (laterality: left hemisphere, right hemisphere) × 3 (frontality: frontal, central, supratemporal) ANOVA was performed for the two deviant stimuli condition (Pattern 1 and Pattern 2) separately. The variance analysis results showed two things. First, in the Pattern 1 condition, none of the main effects or interactions were significant. Second, in the Pattern 2 condition, the differences of the MMN for the GP and PP groups were marginally significant (F(2,26) = 3.66, p = 0.067, GP = -0.80μV, PP = -0.47μV). The interaction of frontality and phonetic discrimination was significant (F(2,26) = 3.38, p = 0.04<0.05). After simple effect inspection, we found that the MMN amplitude for the GP group was larger than the PP group at the central location (t(13) = 2.12, p = 0.044<0.05, GP = -0.86μV, PP = -0.21μV). The difference in the MMN for the GP and PP groups was marginally significant at the frontal location (t(13) = 1.87, p = 0.07, GP = -1.10μV, PP = -0.57μV). The difference in the MMN for the GP and PP groups was not significant at the supratemporal location. The other main effects and interactions were not significant.

When we used the pattern in Díaz et al.’s (2008) study, which we called Pattern 1, we obtained the same results as Díaz et al. (none of the main effects or interactions were significant). According to the analysis of the current study, the differences were not significant might due to the stimuli were too easy to distinguish. Therefore, when we increased the difficulty of the materials for identifying the pure tone pattern based on Pattern 1, which we called Pattern 2, the MMN amplitude for the GP group was significantly larger than for the PP group at the central location. This study indicated that the GP group was better than the PP group in perceiving general acoustic patterns after we increased the difficulty of the materials (i.e., in the Pattern 2 condition), which complied with the assumptions of this research.

## Discussion

### The origin of individual differences in L2 phonetic discrimination

The main goal of this study was to identify the neural correlates of general psychoacoustic abilities and phonetic discrimination in perceiving L2 phonetic contrasts. So we tested whether such individual variability stems from the differences in the general psychoacoustic abilities between L2 good perceivers and poor perceivers. Two groups of early and highly proficient Cantonese (L1)-Mandarin (L2) bilinguals differing in their perception of an L2 phonetic contrast were presented with three general acoustic conditions: frequency, duration, and pattern. As a result, significant differences were obtained between the good perceivers and poor perceivers in all of the three conditions.

Díaz et al.’s (2008) study showed that the individual variability in perceiving L2 phonetic contrasts is linked to the perceivers’ speech-specific capabilities, rather than the perceivers’ general psychoacoustic abilities [1]. In that study, brain activity of Spanish-Catalan bilinguals who differed in their mastery of the Catalan (L2) phonetic contrast /e-ε/ in response to acoustic change detection was recorded in three different conditions (duration, frequency, and presentation order). As a result, participants’ discrimination accuracy, reflected electrically as a mismatch negativity (MMN), was similar between the two groups of participants in the three conditions. Therefore, the results denied the general psychoacoustic origin of individual variability in L2 phonetic mastery. However, the evidence provided by Díaz et al.’s (2008) may be caused by the restricted range of task difficulty. After close analysis of the experiments in Díaz et al.’s (2008) study, We speculated that there were no differences in pure tone duration and frequency discrimination between the good perceivers and poor perceivers might because the stimuli were too difficult for the Spanish-Catalan bilinguals, and no differences in pattern condition might because the stimuli were too easy. Therefore, it is necessary to adjust the difficulty of the task used in Díaz et al.’s (2008) study in order to test the differences in the psychoacoustic abilities of bilinguals between L2 good perceivers and poor perceivers.

This study used Díaz et al.’s (2008) general study design with a modified task. Firstly, in the duration condition, this study recruited early, proficient Cantonese (L1)-Mandarin (L2) bilinguals who are more sensitive to sound duration. Secondly, in the frequency condition, this study lowered the difficulty of the material by extending the duration of pure tone in identifying the pure tone frequency. Thirdly, in the pattern condition, this study increased the difficulty of the material in identifying the pure tone pattern in Díaz et al.’s (2008) study.

This study explored whether the individual differences in perceiving L2 stem from participants’ ability to discriminate various pure tones (frequency, duration and pattern) in the oddball paradigm. The results showed that significant differences between good perceivers (GPs) and poor perceivers (PPs) were found in the three acoustic conditions (frequency, duration and pattern), and the mismatch negativity (MMN) was elicited at frontal and central lobe (F3/ F4 and C3/C4): (1) The MMN was significantly reduced in PP when they were presented with 40ms deviant stimuli in the duration condition. (2) When the duration of pure tone was extended from 50ms to 120ms, the MMN was significantly reduced in PP when they were presented with 1060hz and 1090hz deviant stimuli in the frequency condition. (3) When the pattern stimuli trains were switched from 6-letter pattern to 4-letter pattern, the MMN was significantly reduced in PP in the pattern condition. In a word, the bilinguals who were good in L2 phonetic discrimination obviously had stronger general psychoacoustic abilities than the ones who were poor in L2 phonetic discrimination in all of the three acoustic conditions. It can be suggested that the differences in phonetic discrimination stem from differences in psychoacoustic abilities in learning the sounds of a second language.

Previous studies of individual language acquisition showed that the capacity to identify general sounds in infancy can affect or even predict language acquisition and development. Kuhl’s research [28] showed that infants use computational strategies to detect the statistical and prosodic patterns in language input, and that this leads to the discovery of phonemes and words. In addition, the acoustic analyses in Jusczyk’s study [7] suggest that pitch changes and in some cases durational changes are potential cues that infants might be using to make their discriminations. A series of studies by Benasich et al. also clearly showed that the infant's capacity for processing general acoustic features would directly affect the establishment and development of the language system [9,10,11]. The individual general acoustic processing system and the phonetic system which has already been established will work together to affect the mastery of non-native speech in learning the sounds of a language, not only the native language, but also a second language, and even more. Therefore, differences in phonetic discrimination not only stem from the differences in speech-specific capabilities but also in psychoacoustic abilities in learning the sounds of a language.

Furthermore, L1 and L2 proficiency might share the same neural mechanisms, as Perani and Abutalebi [29] claim that ‘‘L2 seems to be acquired through the same neural devices responsible for L1 acquisition,” based on several neuroimaging and neurophysiological studies on the neural organization of language. This means that the individual general acoustic processing system can affect L1 acquisition, and can also affect L2 acquisition. For example, a series of training studies about L2 consonant learning of adult monolinguals by Golestani et al. [15] found that the psychoacoustic ability of faster phonetic learners to process rapidly changing stimuli was better than that of slower phonetic learners, suggesting that morphological correlates of phonetics were related to the ability to process rapid temporal variation. The most distinguishing features of consonant speech are the discontinuity and the rapid change of sound, so the research suggested that the differences of phonetic acquisition stem from the individual differences in learning the phonetic acoustic features. Their subsequent research further found that left auditory cortex WM anatomy, which likely reflects auditory processing efficiency, partly predicts individual differences in an aspect of language learning that relies on rapid temporal processing [16,17]. In a word, the phonetic training studies suggest that the psychoacoustic abilities could affect phonetic learning. In another word, they supported that the differences in phonetic discrimination stem from the differences in psychoacoustic abilities in learning the sounds of a second language.

It should be noted that this study used a correlational design, and the results only showed the correlation between speech perception and psychoacoustic abilities of the bilinguals in their second language. However, combined with the relevant research results of preceding studies, we can infer that psychoacoustic abilities affect L2 phonetic discrimination, in another word, differences in phonetic discrimination stem from the differences in psychoacoustic abilities in learning the sounds of a second language.

### The relationship of individual native language and psychoacoustic ability

The language environment of early childhood guides the development of highly accurate phonetic categorization and discrimination [18,28]. In most languages, general acoustic information supplementary to phonetic segments (or phones) is available to distinguish different meanings in words. For instance, duration gives rise to a phonological contrast in the form of length or quantity. In quantity languages, phoneme length can distinguish between different meanings of otherwise similar words. In Cantonese, changing the length of /a/ in the word /gap3/ (夹) will lead to word / gaap3 / (甲). In terms of acoustic variance, these duration differences can be very subtle, the duration of short phones ranging between a few 10s and over 100ms. Thus, quantity-language speakers are more sensitive not only to the phonetic duration but also to the acoustic duration than the speakers of a non-quantity language. In addition, previous research showed that quantity-language speakers were superior to speakers of a non-quantity language in discriminating the duration of non-speech sounds [30].

Therefore, it is worth noting that in this study the participants were Cantonese (L1)-Mandarin (L2) bilinguals, while in Díaz et al.’s (2008) study the participants were Spanish (L1)-Catalan (L2) bilinguals. As we known, Spanish is a non-quantity language, but Cantonese is a quantity language. Specifically, in Spanish, there are five vowels: A\E\I\O\U(no distinction between the duration), while in Cantonese, there are seven long vowels: A\E\I\O\U\Eo\Eu and three short vowels: Ah\Eh\Oh. Which means that vowel durations are more important for Cantonese than for Spanish listeners. Thus, we would assume that the native Cantonese speakers in this study would be superior to the native Spanish speakers in Díaz et al.’s (2008) study in discriminating the duration of non-speech. The results of this study were consistent with this idea: the deviant stimuli 40ms and 80ms both elicited reliable MMN for the GP and PP groups of the native Cantonese speakers, while only the larger deviant stimuli (40ms) elicited a reliable MMN for the GP group of the native Spanish speakers in Díaz et al.’s (2008) study. Thus it can be seen that individual native language will affect general acoustic discrimination.

**Fig 4:**
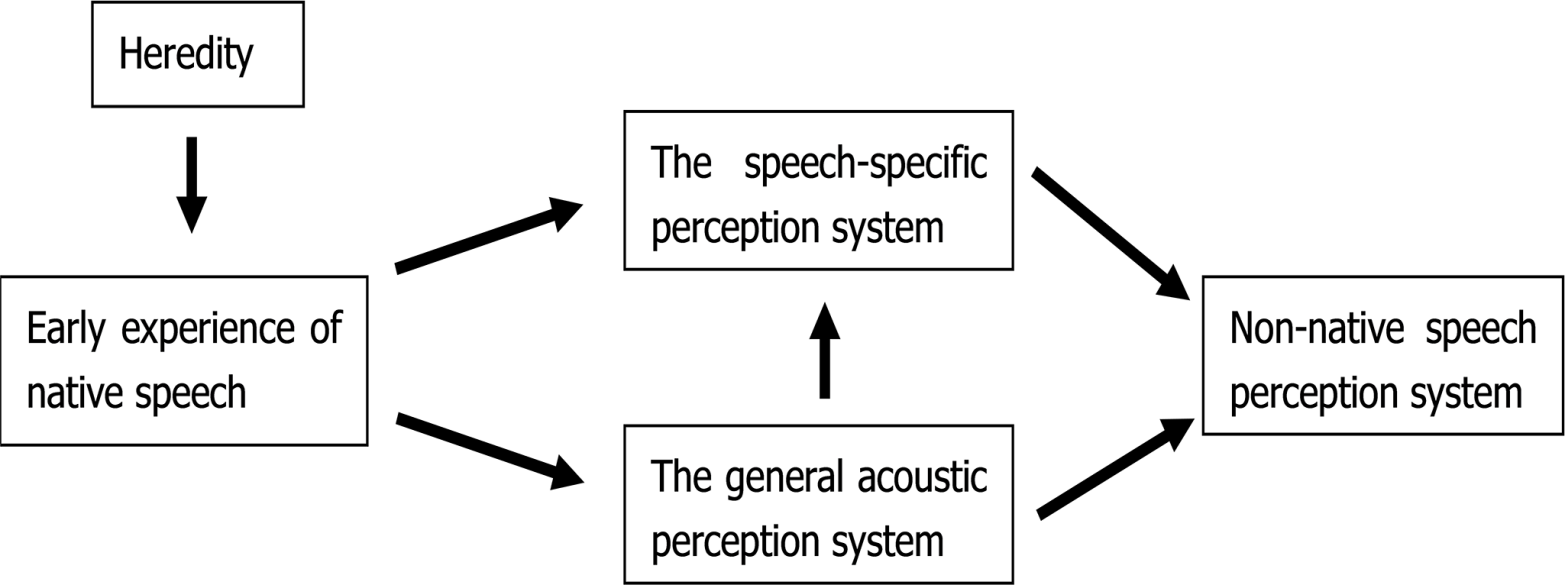
Sources of difference in individual speech discrimination.

The underlying reason for this phenomenon is that the early experience of native speech will influence the formation of not only the infant's speech perception system, but also the general acoustic perception system. Native language experience in the infancy stage involves some pronunciations appearing frequently in the mother tongue; because infants use computational strategies to detect the statistical and prosodic patterns in language input, leading to the discovery of phonemes and words [28], such experience of learning native speech will affect the sensibility of infants to the acoustic features included in speech. Kuhl’s (2004) research showed that infants’ inborn acoustic sensitivities varied based on differences in psychoacoustic abilities: Infants were not only able to acquire speech using statistical cues (such as speech occurrence and distributional frequencies), but also able to develop sensitivity to some acoustic features, especially, the acoustic features that frequently occurred in native speech. In other words, the early experience of native speech not only influences the development of an individual speech-specific perception system, but the acoustic features included in native speech also form an individual general acoustic perception system. Thus it can be seen that early experience of native speech plays a very important role in the acquisition and mastery of a second language. On the one hand, early experience of native speech will directly influence the development of an individual speech-specific perception system; on the other hand, early experience of native speech will influence the development of an individual general acoustic perception system, which will influence language development (see Fig 4).

Strictly speaking, it is possible that there are many more reasons according to which two measures can be correlated. For instance, it is possible that the good perceivers were more motivated participants (e.g., paying more attention in the experiments), hence they were better at phonetic discrimination on the one hand and the acoustic tasks on the other. In further research, we will explore more relevant factors which influence the individual difference in L2 phonetic discrimination and the relationship with psychoacoustic abilities.

## Conclusion

The results of this study showed that differences in L2 phonetic discrimination were related to the differences in general psychoacoustic abilities. Individuals who were different in L2 phonetic discrimination (good perceivers and poor perceivers) had significant differences in discriminating various pure tones (frequency, duration and pattern). Therefore, combined with the earlier theoretical studies, we can suggest that differences in phonetic discrimination stem from differences in psychoacoustic abilities in learning the sounds of a second language.

## Supporting information

S1 File Experiment data of duration condition. (RAR)

S2 File Experiment data of frequency condition. (RAR)

S3 File Experiment data of pattern condition. (RAR)

## References

[1] Strange W., Dittman S. (1984) Effects of discrimination training on the perception of /r-l/ by Japanese adults learning English. Percept Psychophys 36: 131–145. 6514522 10.3758/bf03202673

[2] Molfese D. L. (2000) Predicting dyslexia at 8 years of age using neonatal brain responses. Brain and Language 72: 238–245. doi: 10.1006/brln.2000.2287 10764519 10.1006/brln.2000.2287

[3] Molfese D. L., Molfese V. J., & Modglin A. (2004) Development of auditory event-related potentials in young children and relations to word-level reading abilities at age 8 years. Annals of Dyslexia 54: 9–38. 15765002 10.1007/s11881-004-0002-3 PMC2729145

[4] Maurer U., Bucher K., Brem S., & Brandeis D. (2003) Altered responses to tone and phoneme mismatch in kindergartners at familial dyslexia risk. NeuroReport 14: 2245–2250. doi: 10.1097/01.wnr.0000096518.69073.a7 14625456 10.1097/00001756-200312020-00022

[5] Meng X., Sai X., Wang C., Wang J., Sha S., & Zhou X. (2005) Auditory and speech processing and reading development in Chinese school children: Behavioural and ERP evidence. Dyslexia 11: 292–310. 16355749 10.1002/dys.309

[6] Jusczyk P.W., Hirsh-Pasek K., Nelson D.G., Kennedy L.J., Woodward A., Piwoz J. (1992) Perception of acoustic correlates of major phrasal units by young infants. Cogn. Psychol. 24 (2): 252–293. 1582173 10.1016/0010-0285(92)90009-q

[7] Kraus N., McGee T.J., Carrell T.D., Zecker S.G., Nicol T.G., Koch D.B. (1996) Auditory neurophysiological responses and discrimination deficits in children with learning problems. Science 273: 971–973. 8688085 10.1126/science.273.5277.971

[8] Benasich A.A., Leevers H.J. (2002) Processing of rapidly presented auditory cues in infancy: implications for later language development. Progress in Infancy Research 3: 245–288.

[9] Benasich A.A., Choudhury N., Friedman J.T., Realpe-Bonilla T., Chojnowska C., Gou Z. (2006) The infant as a prelinguistic model for language learning impairments: predicting from event-related potentials to behavior. Neuropsychologia 44: 396–411. doi: 10.1016/j.neuropsychologia.2005.06.004 16054661 10.1016/j.neuropsychologia.2005.06.004 PMC1569769

[10] Choudhury N., Benasich A. (2010) Maturation of auditory evoked potentials from 6 to 48 months: prediction to 3 and 4 year language and cognitive abilities. Clin. Neurophysiol: 122(2): 320–338. doi: 10.1016/j.clinph.2010.05.035 20685161 10.1016/j.clinph.2010.05.035

[11] Telkemeyer S., Rossi S., Koch S.P., Nierhaus T., Steinbrink J., Poeppel D., et al (2009) Sensitivity of newborn auditory cortex to the temporal structure of sounds. J. Neurosci. 29 (47): 14726–14733. doi: 10.1523/JNEUROSCI.1246-09.2009 19940167 10.1523/JNEUROSCI.1246-09.2009 PMC6666009

[12] Tallal P., Miller S., Fitch R.H. (1993) Neurobiological basis of speech: a case for the preeminence of temporal processing. Annals of the New York Academy of Sciences 682(1):27–47. 7686725 10.1111/j.1749-6632.1993.tb22957.x

[13] Wright B.A., Lombardino L.J., King W.M., Puranik C.S., Leonard C.M., Merzenich M.M. (1997) Deficits in auditory temporal and spectral resolution in language-impaired children. Nature 387: 176–178. doi: 10.1038/387176a0 9144287 10.1038/387176a0

[14] Golestani N, Paus T, Zatorre RJ (2002) Anatomical correlates of learning novel speech sounds. Neuron 35: 997–1010. 12372292 10.1016/s0896-6273(02)00862-0

[15] Golestani N, Zatorre RJ (2004) Learning new sounds of speech: Reallocation of neural substrates. NeuroImage 21: 494–506. doi: 10.1016/j.neuroimage.2003.09.071 14980552 10.1016/j.neuroimage.2003.09.071

[16] Golestani N, Molko N, Dehaene S, LeBihan D, Pallier C (2007) Brain structure predicts the learning of foreign speech sounds. Cereb Cortex 17: 575–582. doi: 10.1093/cercor/bhk001 16603709 10.1093/cercor/bhk001

[17] Díaz B., Baus C., Escera C, Costa A., Sebastian-Galles N. (2008) Brain potentials to native phoneme discrimination reveal the origin of individual differences in learning the sounds of a second language. PNAS 105(42): 16083–16088. doi: 10.1073/pnas.0805022105 18852470 10.1073/pnas.0805022105 PMC2570969

[18] Näätänen R. (2001) The perception of speech sounds by the human brain as reflected by the Mismatch Negativity (MMN) and its magnetic equivalent (MMNm). Psychophysiology 38: 1–21. 11321610 10.1017/s0048577201000208

[19] Lang H, Nyrke T, Ek M, Aaltonen O, Raimo I, Näätänen R (1990) Pitch discrimination performance and auditory event-related potentials. Psychophysiol Brain Res 1: 294–298.

[20] Näätänen R, Lehtokoski A, Lennes M, Cheour M, Huotilainen M, Iivonen A, et al (1997) Language-specific phoneme representations revealed by electric and magnetic brain responses. Nature 385: 432–434. doi: 10.1038/385432a0 9009189 10.1038/385432a0

[21] Nenonen S, Shestakova A, Huotilainen M, Näätänen R (2005) Speech-sound duration processing in a second language is specific to phonetic categories. Brain Lang 92: 26–32. doi: 10.1016/j.bandl.2004.05.005 15582033 10.1016/j.bandl.2004.05.005

[22] Tremblay K, Kraus N, McGee T (1998) The time course of auditory perceptual learning: Neurophysiological changes during speech-sound training. NeuroReport 9: 3557–3560. 9858359 10.1097/00001756-199811160-00003

[23] Winkler I, Kujala T, Tiitinen H, Sivonen P, Alku P, Lehtokoski A, et al (1999) Brain responses reveal the learning of foreign language phonemes. Psychophysiology 36: 638–642. 10442032

[24] Näätänen R., Gaillard A.W.K., Mäntysalo S. (1978). Early selective-attention effect on evoked potential reinterpreted. Acta Psychol. 43: 313–329. 10.1016/0001-6918(78)90006-9 685709

[25] Näätänen R., Jacobsen T., Winkler I. (2005) Memory-based or afferent processes in mismatch negativity (MMN): a review of the evidence. Psychophysiology 42: 25–32. doi: 10.1111/j.1469-8986.2005.00256.x 15720578 10.1111/j.1469-8986.2005.00256.x

[26] Sebastián-Gallés N, Baus C (2005) On the relationship between perception and production in L2 categories. In Twenty-first Century Psycholinguistics: Four Cornerstones, ed Cutler A (Erlbaum, New York): 279–292.

[27] McNicol D. (2005) A primer of signal detection theory: Psychology Press.

[28] Kuhl P. (2004) Early language acquisition: Cracking the speech code. Nature Reviews Neuroscience 5: 831–843. doi: 10.1038/nrn1533 15496861 10.1038/nrn1533

[29] Perani D, Abutalebi J (2005) The neural basis of first and second language processing. Curr Opin Neurobiol 15: 202–206. doi: 10.1016/j.conb.2005.03.007 15831403 10.1016/j.conb.2005.03.007

[30] Tervaniemi M, Jacobsen T, Röttger S, Kujala T, Widmann A, Vainio M, et al (2006) Selective tuning of cortical sound-feature processing by language experience. Eur J Neurosci 23: 2538–2541. doi: 10.1111/j.1460-9568.2006.04752.x 16706861 10.1111/j.1460-9568.2006.04752.x

