# Correction: Differences in phonetic discrimination stem from differences in psychoacoustic abilities in learning the sounds of a second language: Evidence from ERP research

**DOI:** 10.1371/journal.pone.0209302

**Published:** 2018-12-12

**Authors:** Yi Lin, Ruolin Fan, Lei Mo

There are passages in the article [1] with text overlap from a previously published article by Diaz et al entitled “Brain potentials to native phoneme discrimination reveal the origin of individual differences in learning the sounds of a second language” in *Proc Natl Acad Sci* USA 2008; 105(42): 16083–8 [2] which was cited in the *PLOS ONE* article [1] as reference 17. The authors have rephrased the text and provided in-text citations to the original study [2] upon which theirs is based. Please see the original text on the left and the author corrected text on the right here:

**Table:** 

**Original text**	**Corrected text**
“There are two alternative explanations: a general psychoacoustic origin vs. a speech specific one” (pp. 1)	“There are two contrast views: general psychoacoustic origin vs. speech-specific origin [17]”
“For example, most Japanese native speakers may consider the English words /rock/ and /lock/ to be the same word, because the Japanese speech-sound system does not distinguish between the phonemes /r/ and /l/ [1].” (pp. 2)	“. For example, most Japanese native speakers may consider the English words /roll/ and /loll/ to be the same word, because the Japanese language system hardly can distinguish between the phonemes /r/ and /l/ [1,17].”
“Furthermore, that research had explored the neural differences between monolinguals trained to differentiate a difficult Hindi (L2) retroflex phonetic contrast.” (pp.2)	“In addition, that research also explored neural differences between monolinguals who were trained to distinguish indistinguishable Hindi (L2) retroflex phonetic contrast. [17]”
“In addition, neural correlates of speech change detection were also assessed for both native (/o/-/e/) and non-native (/o/-/ö /) phonetic contrasts (speech condition). Participants’ discrimination accuracy, reflected electrically as a mismatch negativity (MMN), was similar between the two groups of participants in the three conditions. Conversely, the MMN was reduced in poor perceivers (PP) when they were presented with speech sounds. Therefore, the results supported a speech-specific origin of individual variability in L2 phonetic mastery.” (pp. 2)	“Furthermore, neural correlations were also assessed for the detection of speech changes between native (/o/-/e/) and non-native (/o/-/ö /) speech contrast (speech condition). Under the three conditions of pure tone, the discrimination accuracy reflected by MMN of the participants was similar between the two groups. On the contrary, when they were presented with speech sounds, MMN decreased in poor perceivers. Therefore, the results supported that the origin of individual differences in learning the sounds of a second language was speech-specific capabilities. [17]”
“…the MMN is elicited when the auditory perceptual system detects a mismatch between a neural representation of a frequently repeated stimulus (the standard) and a stimulus deviating in at least one parameter (the deviant). This ERP component peaks between 100–250ms.” (pp. 4)	“…MMN is triggered when the auditory perception system detects a mismatch between the neural representation of frequent repetitive stimuli (standard) and stimuli that deviate from at least one parameter (deviation). The peak of this ERP component is between 100 and 250ms. [17]”
“Therefore, it is supposed that the neural activity underlying the MMN is attributable to two sets of neural generators: a superior temporal and a fronto-central generator. The former is associated with processing the auditory sensory input against a formed memory trace, whereas the latter is related to an involuntary attention switch toward a detected change in the auditory input [24,25]. Thus…” (pp. 4)	“According to previous studies, in the auditory input, the fronto-central area is associated with an involuntary attention switch toward a detected change, whereas the superior temporal area is related to the processing of auditory sensory input against a formed memory trace [17,24,25]. Therefore…”
“…and none of them reported having any hearing or language difficulties or receiving specific musical training.” (pp. 6)	“… Each participant had no hearing or language difficulties, and no one received specific music training. [17]”
“In experiment 1, we used the same sound stimuli as in Díaz et al.’s (2008) study.” (pp. 6)	“Our methodology was based on Diaz et al.’s article. In experiment 1, we used the same sound stimuli as in Díaz et al.’s (2008) study. [17]”
“In the duration condition, the stimuli were pure tones of 1,000 Hz. The duration of the standard tone was 200ms (including 10ms of rise/fall times) and the durations of the three deviant tones were 120, 80, and 40ms.” (pp. 6)	“The stimuli were pure tones of 1,000 Hz in the duration condition. The duration of the standard tone was 200ms, while the durations of three deviant tones were 40, 80, and 120ms. [17]”
“The frequency of the standard tone was 1,000 Hz, whereas the frequencies of the deviant tones were 1,030, 1,060, and 1,090 Hz. The probability of the standard tone was always 0.8 (600 standard tones per block), and for each deviant tone the probability was 0.066 (50 presentations of each deviant tone per block). Tones were presented in random order with the restriction that the first five tones of the blocks were always a standard and that at least one standard tone was presented between two deviants. The SOA was314ms.” (pp. 6)	“The frequency of the standard tone was 1,000 Hz, whereas the frequencies of the deviant tones were 1,090, 1,060, and 1,030 Hz. The probability of the standard tone was always 0.8 (600 standard tones per block), and the probability was 0.066 (50 presentations of each deviant tone per block) for each deviation. The tones were presented in random order. The limitation was that the first five tones of a block were always standard and presented at least one standard tone between two deviations. The SOA was 314ms. [17]”
Each train consisted of six alternating pure tones of either 500 or 1,000 Hz (2,400 tones altogether). Tones lasted 50ms, including 10ms rise/fall times. Tones within and between the trains were presented at a constant SOA of 128ms. Stimuli trains were presented in a predictable way (ABABAB-BABABA-BABABA-ABABAB…),in which A represents the 500 Hz tone and B the 1,000 Hz tone, the hyphen indicates the beginning of the trains, and the bolded A and B denote the deviant event.” (pp. 6)	“Each train was constituted by six alternating pure tones of either 500 or 1000 Hz (2,400 tones altogether). Tones lasted 50ms (including 10ms rise/fall times) and were presented at a constant SOA of 128ms within and between the trains. Stimuli trains were presented in a pattern (ABABAB-BABABA-BABABA-ABABAB…), in which A represented the 500 Hz tone and B represented the 1,000 Hz tone. [17]”
“The ERPs were recorded from the scalp by using tin electrodes mounted in an electrocap (Electro-Cap International) and located at six standard positions (F3, Fz, F4, C3, Cz, and C4) and the two mastoids (LM and RM).” (pp. 7)	“The ERPs were recorded at six standard positions (F3, Fz, F4, C3, Cz, and C4) and the two mastoids (LM and RM) of the scalp by using an electrocap. [17]”
“Eye movements were measured with electrodes attached to the infraorbital ridge and on the outer canthus of the right eye. The common EEG/electrooculogram (EOG) reference was attached to the tip of the nose. Electrode impedances were kept <5kΩ. The electrophysiological signals were filtered on-line with a bandpass of 0.1–100 Hz and digitized at a rate of 1000 Hz.” (pp. 7)	“Eye movements were measured by electrodes attached to the infraorbital ridge and the lateral canthus of the right eye. The common electroencephalograph reference was attached to the tip of the nose. The electrode impedance was kept <5kO. The electrophysiological signals were digitized at a rate of 1000 Hz and filtered on-line with a bandpass of 0.1 to 100 Hz. [17]”
“Epochs included in all cases a prestimuli baseline of 100ms and were 600ms long. Baseline was corrected, and the linear DC detrend procedure was performed on the individual segments.” (pp. 7)	“The duration of stimulation was 600ms included a prestimuli baseline of 100ms. The baseline was corrected, and the linear DC detrended procedures were carried out on all segments. [17]”
“The MMN was measured for each participant group and condition as the mean amplitude in a 20ms latency window centered at its maximum peak. To test whether a significant MMN was elicited by deviant stimuli, one sample t-tests were carried out (separately for each group of participants) to compare the amplitudes of the MMN component at Fz against the zero level [1].” (pp. 7)	“MMN of each participant group and condition was measured as the average amplitude in the 20 ms latency window centered on the maximum peak. One sample t-tests (for each group of participants) were conducted to compare the magnitude and zero level of MMN components at Fz to test whether deviating stimuli induced significant MMN. [17]”
“Only deviants eliciting a reliable MMN at least for one group were included in the analysis. In this step, the positive MMN for the supratemporal lobe had to be reversed into negative MMN with the same absolute value. The factor’s laterality (left hemisphere: F3, C3, LM; right hemisphere: F4, C4, RM), frontality (frontal location: F3, F4; central location: C3, C4; supratemporal location: LM, RM), and deviant type (when necessary) were included in the ANOVA as within subjects factors, whereas participant group (GPs and PPs) was the between subjects factor.” (pp. 7)	“Only the deviant can produce a reliable MMN for at least one group were included in the analysis. In this step, the positive MMN in the temporal lobe must be inverted to negative MMN with the same absolute value. The lateral position factor (left hemisphere: F3, C3, LM; right hemisphere: F4, C4, RM), frontality factor (frontal location: F3, F4; central location: C3, C4; superatemporal location: LM, RM), and deviant type as within subjects factors were included in the ANOVA, whereas participant group was the between subjects factor. [17]”
“So we tested whether such individual variability stems from the differences in the general psychoacoustic abilitiesbetweenL2 good perceivers and poor perceivers.” (pp. 12)	“So we examined the individual differences originates in the general psychoacoustic abilities. [17]”
